# MaTrICS: Micromobility Associated With Trauma and Its Clinical and Socioeconomic Impact

**DOI:** 10.7759/cureus.100758

**Published:** 2026-01-04

**Authors:** Rita Leite Cruz, Joana Alves Cabrita, Rui Caetano Garcês, Mafalda Gama, André Oliveira, Ricardo Júnior, Rui Cunha, Lúcia Proença, Diogo Lopes, Sara Machado, Simão C Rodeia, João Melo Alves, Luis Bento

**Affiliations:** 1 Intensive Care Medicine, Local Health Unit São José, Lisbon, PRT; 2 Intensive Care Medicine, Local Health Unit Estuário do Tejo, Lisbon, PRT; 3 Intensive Care Medicine, Local Health Unit Lisboa Ocidental, Lisbon, PRT; 4 Intensive Care Medicine, Hospital de São José, Lisbon, PRT

**Keywords:** clinical and socioeconomic impact, critical care, critical patient, major trauma, micromobility associated

## Abstract

Micromobility includes lightweight vehicles, such as bicycles and electric scooters, with limited speed and weight, and is promoted as a sustainable alternative for urban transport. Its expansion has been accompanied by a significant rise in accidents, particularly among users without helmets or under the influence of alcohol. In Portugal, despite growing adherence, data on the clinical and economic impact of micromobility remain scarce. This study aims to characterise the morbidity, severity, and associated costs of these accidents in a Portuguese tertiary hospital between 2019 and 2022.

A retrospective observational study was conducted at Unidade Local de Saúde de São José, including adult patients with trauma related to micromobility vehicles between January 2019 and December 2022. Data were retrieved from hospital clinical records. Drivers or occupants of motorcycles were excluded. Statistical analysis was performed using IBM SPSS® (IBM Corp., Armonk, NY), applying the Mann-Whitney test (p < 0.05).

A total of 1,566 patients with micromobility-related trauma were included, mostly from single-vehicle incidents (n = 1,379; 88.1%) and involving electric scooters (n = 1,015; 64.8%). The mean age was 50 years, and 677 patients (43.2%) were Portuguese nationals. Helmet use was reported in only 21 of 302 cases with available data (7%). The most frequent injuries affected the limbs (n = 1,000; 63.8%) and the head (n = 718; 36.9%), with a mean Injury Severity Score (ISS) of 2.7. Sixteen cases (1.0%) were classified as severe (ISS ≥ 16). Alcohol consumption was significantly associated with greater severity (p = 0.009). A total of 271 patients (17.3%) were hospitalised, 18 of whom (1.1%) required intensive care. The estimated total cost of hospitalisation was €456,000. There were five in-hospital deaths (0.3%) and 34 cases (2.2%) of persistent neurological sequelae.

Most micromobility accidents involved scooters and resulted from single-vehicle crashes, reflecting patterns similar to those reported internationally. Risk behaviours, such as low helmet use and alcohol consumption, were identified, the latter being associated with increased injury severity. Although minor injuries predominated, a subgroup sustained severe trauma with significant clinical, functional, and economic impact. These findings highlight the need for preventive strategies and standardised registries to support effective monitoring and the implementation of targeted public health measures.

Although minor injuries were most frequent, a minority of cases involved severe trauma with relevant clinical and economic impact. The findings support the need for preventive measures and prospective data collection systems to inform effective public policies.

## Introduction

Micromobility was defined in 2018 by the Society of Automotive Engineers (SAE) International as a class of vehicles with a gross weight under 500 kg, without internal combustion engines, and with a maximum speed below 45 km/h. This category includes individual transport modes -motorised or non-motorised - designed for short distances and limited speeds, such as conventional or electric bicycles, electric scooters, and monowheels [1].

At the European level, the promotion of micromobility is part of broader goals for sustainable urban mobility, aligned with the United Nations’ Sustainable Development Goals for 2030. In recent years, urban mobility has undergone a significant transformation, with e-mobility - understood as the use of electrically powered vehicles, such as electric bicycles and scooters, integrated into digital urban transport solutions - emerging as an accessible, efficient, and environmentally sustainable alternative [2].

In Portugal, public micromobility was first introduced in 2001, with the launch of a free bicycle-sharing service in Cascais. From 2017 onward, there was a marked expansion of electric scooter and bicycle operators in urban settings. In parallel, the legal framework for these vehicles was established in 2013 and revised in 2021, legally equating electric bicycles and electric scooters, requiring their circulation on public roads and recommending - though not mandating - helmet use [3-4].

International studies have reported a significant increase in traumatic injuries related to micromobility. These findings highlight not only rising incidence but also clinically relevant injury severity, with several factors associated with worse outcomes (e.g., lack of helmet use, collisions at intersections, unsafe road conditions, and behavioural factors, such as alcohol consumption). Zhao et al., in an age-stratified analysis conducted between 2015 and 2019 across China, India, Japan, and the United States, found an increased mortality risk among users over 45 years of age [5]. In Spain, Sanjurjo-de-No et al. analysed approximately 6,000 accidents between 2016 and 2020, concluding that not wearing a helmet, excessive speed, poor road conditions, and crashes at intersections were strongly associated with severe injuries [6].

In Portugal, the trend appears to mirror the international scenario. According to the 2023 Annual Road Safety Report, 3,239 accidents involving bicycles and scooters were recorded, representing a 38.2% increase compared to 2019 [7].

Given the lack of national data on this topic, we conducted this study within the Micromobility Associated with Trauma and its Clinical and Socioeconomic Impact (MaTrICS) project, in a Portuguese tertiary hospital (2019-2022). The primary objective was to describe the clinical profile and injury severity of micromobility-related trauma. Secondary objectives were to quantify healthcare resource utilisation and socioeconomic impact, and to assess factors associated with increased injury severity and resource use, including helmet use and alcohol consumption.

## Materials and methods

This was a retrospective, observational, and non-interventional study conducted at São José Local Health Unit (Unidade Local de Saúde de São José), involving adult patients admitted for trauma related to micromobility vehicles between January 2019 and December 2022.

**Figure 1:**
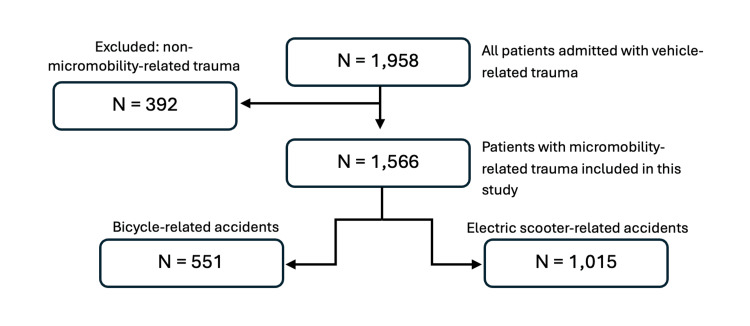
Flowchart of patient selection. Among 1,958 patients admitted with vehicle-related trauma, 392 with non-micromobility-related injuries were excluded, leaving 1,566 patients with micromobility-related trauma, who were categorised into bicycle-related (n = 551) and electric scooter-related (n = 1,015) accidents.

Data were collected through review of Emergency Department (ED) clinical records at Unidade Local de Saúde de São José. Each patient was counted only once. During the study period, 1,958 patients presented to the ED with vehicle-related trauma; 392 with non-micromobility-related injuries, including drivers or passengers of motorcycles, were excluded. The final cohort comprised 1,566 victims of accidents involving micromobility vehicles (conventional or electric bicycles and scooters) who presented to the ED and were categorised into bicycle-related (n = 551; 35.2%) and electric scooter-related (n = 1,015; 64.8%) accidents, as shown in Figure 1.

Missing data were expected due to the retrospective design. No imputation was performed. Analyses were conducted using available data for each variable (complete-case per analysis), and denominators are reported accordingly; when information was not recorded in the ED chart, the variable was classified as missing/unknown.

Toxicology testing (e.g., blood alcohol concentration and/or urine drug screening) was not performed systematically; it was requested at the discretion of the treating clinician based on clinical judgment and ED protocols. Alcohol and drug use were coded as positive when there was a documented positive test and/or explicit clinical documentation; in the absence of testing or documentation, the status was recorded as unknown.

Statistical analysis was performed using IBM SPSS Statistics (version 30.0.0.0, IBM Corp., Armonk, NY). Continuous variables not following a normal distribution were compared using the Mann-Whitney U test. A p-value < 0.05 was considered statistically significant.

For analysis purposes, motor sequelae were defined as patient-reported alterations in muscle strength, while neurological sequelae referred to sensory, coordination, balance, speech, communication, or cognitive impairments reported by the patients.

Trauma severity was assessed using the Injury Severity Score (ISS), an anatomical score in which severe trauma is commonly defined as ISS ≥ 16. For patients admitted to the ICU, the severity of critical illness was evaluated with the Acute Physiology and Chronic Health Evaluation II (APACHE II) score and the Simplified Acute Physiology Score (SAPS II/SAPS III), which are widely used prognostic scores in intensive care [8-11].

Cost estimates were calculated from the hospital/provider perspective and represent direct medical costs limited to inpatient stay (general ward and ICU) and CT imaging. Unit costs were obtained from the applicable national ordinances and applied as follows:

(i) general ward cost = number of hospitalized patients × length of stay (days) × daily ward tariff;

(ii) ICU cost = number of ICU admissions × ICU length of stay (days) × daily ICU tariff; and

(iii) CT cost = total number of CT scans × unit tariff per CT.

Costs were reported in euros and were not adjusted for inflation; ED costs, laboratory tests, medications, surgical procedure costs, and post-discharge costs were not included. Different ordinances were used because they provide the most recent official tariffs available for each cost component at the time of analysis.

The study was approved by the Ethics Committee of ULS São José (reference CA_INV 474) and was conducted in full compliance with the General Data Protection Regulation (GDPR).

## Results

Sample characterization

**Figure 2:**
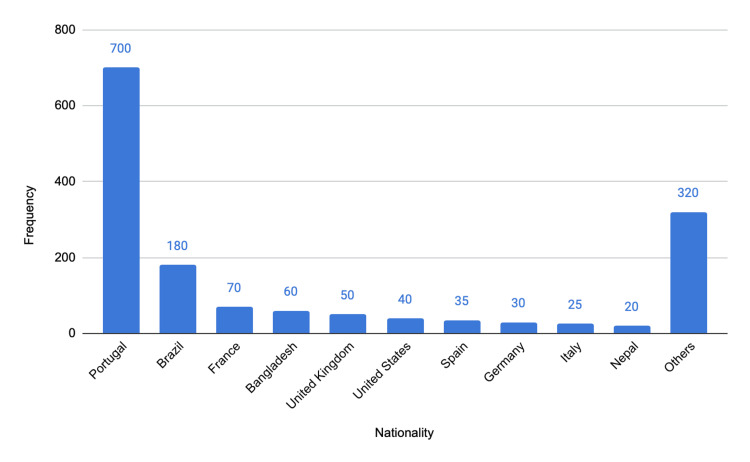
Geographical distribution of the 10 most frequent nationalities. The y-axis represents numbers.

A total of 1,566 patients admitted between January 2019 and December 2022 for trauma of any severity associated with micromobility vehicles were included. The mean age was 50 ± 14.2 years, and 43.2% (n = 677) were Portuguese nationals (Figure 2).

When analysing age by type of vehicle, scooter-related accidents occurred in younger patients (35.3 ± 11.9 years, median 32) compared to bicycle-related accidents (42.0 ± 16.4 years, median 38) (Table 1). There were no statistically significant differences in APACHE II, SAPS II, or SAPS III between the groups, indicating that they were comparable in terms of baseline severity.

**Table 1:** Baseline characteristics of patients with micromobility-related trauma according to type of vehicle. Data are presented as mean ± standard deviation for continuous variables and n (%) for categorical variables, unless otherwise indicated. APACHE II, Acute Physiology and Chronic Health Evaluation II; ICU, intensive care unit; ISS, Injury Severity Score; SAPS II, Simplified Acute Physiology Score II; SAPS III, Simplified Acute Physiology Score III; TBI, traumatic brain injury

Variable	Global	Electric Scooters	Bicycles
Total patients	1,566	1,015 (64.8%)	551 (35.2%)
Mean age	50 ± 14.2	35.3 ± 11.9	42.0 ± 16.4
Portuguese nationality	677 (43.2%)	387 (38.1%)	290 (52.6%)
Single-vehicle crash	1,379 (88.1%)	929 (91.5%)	450 (81.7%)
Pedestrian hit	97 (6.2%)	51 (5%)	46 (8.3%)
Collision with vehicle	83 (5.3%)	33 (3.3%)	50 (9.1%)
Mean ISS	2.7 ± 3.7	2.52 ± 2.8	3.0 ± 5.1
Severe trauma (ISS ≥ 16)	16	10	6
Limb injuries	1,000 (63.8%)	631 (62.2%)	369 (67%)
Head trauma (TBI)	718 (36.9%)	493 (48.6%)	225 (40.8%)
Thoracoabdominal injuries	167 (8.6%)	93 (9.2%)	74 (13.4%)
Spinal cord injuries	60 (3.1%)	29 (2.9%)	31 (5.6%)
Hospital admission	271 (17.3%)	109 (10.7%)	162 (29.4%)
ICU admission	18 (1.1%)	10 (1.0%)	8 (1.5%)
Mean APACHE II	11.1	11.1	11
Mean SAPS II/SAPS III	35.6 / 57.5	35.6 / 57.5	35.6/57.5

Accident pattern

**Figure 3:**
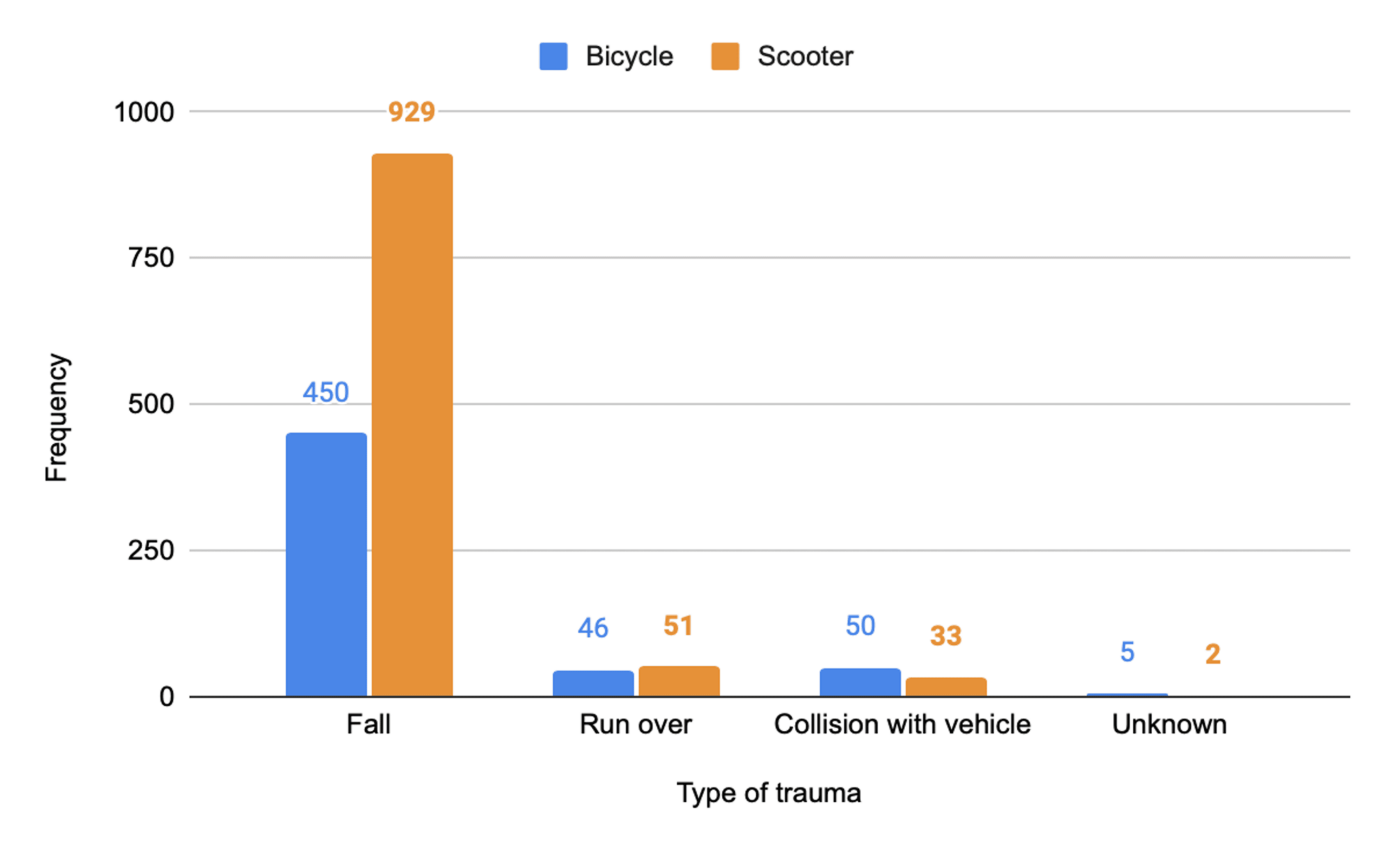
Distribution by type of trauma and micromobility vehicle in the general population. The y-axis represents numbers.

**Figure 4:**
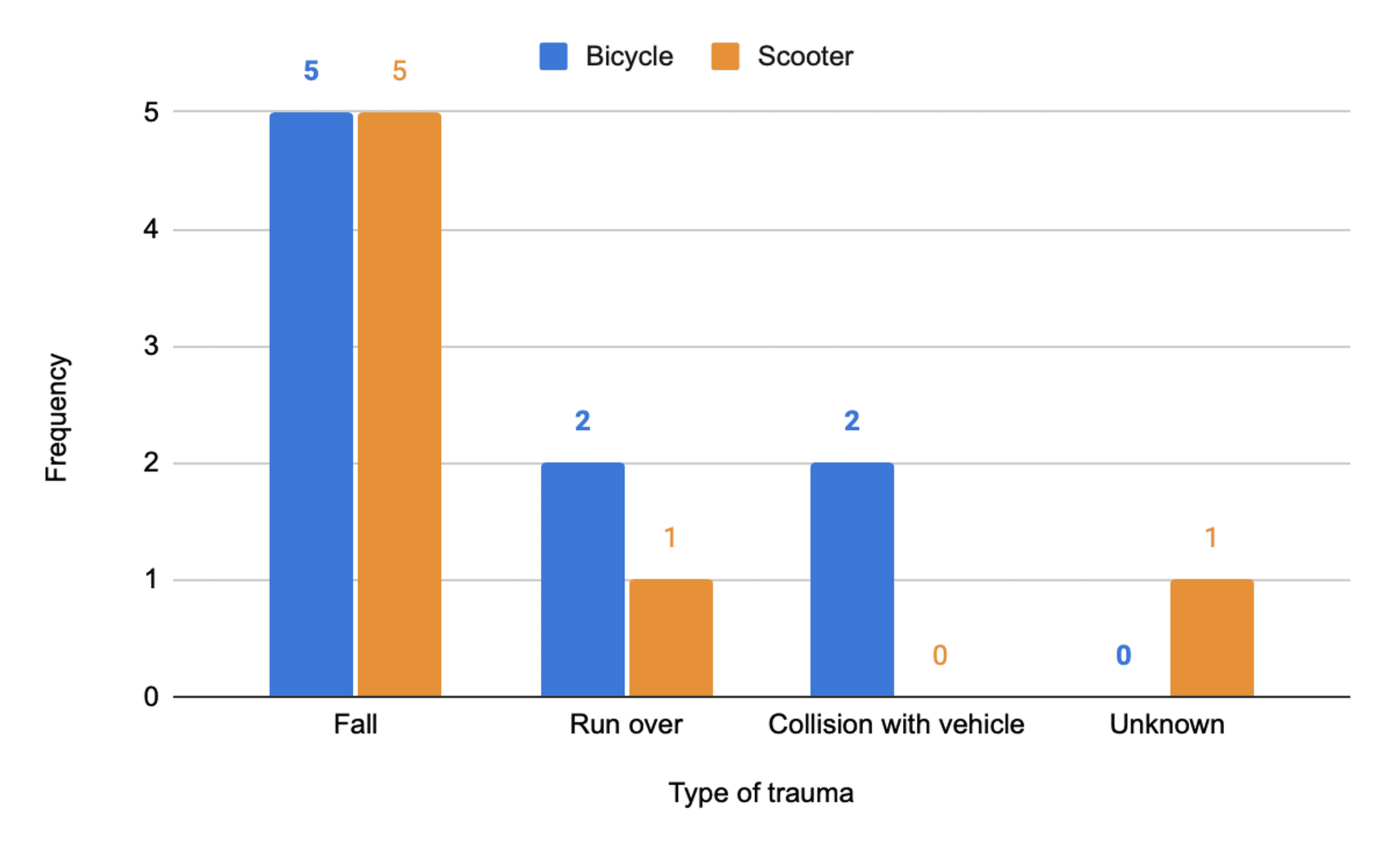
Distribution by type of trauma and micromobility vehicle in the general population in the severe subgroup (ISS ≥ 16). ISS, Injury Severity Score The y-axis represents numbers.

The majority of accidents involved e-scooters. Overall, 88.1% (N = 1,379) of incidents resulted from single-vehicle crashes without third-party involvement, with more than half (N = 929; 59.3%) associated exclusively with e-scooter use. Among e-scooter users, 91.5% (N = 929) of accidents were due to loss of control, compared to 81.7% (N = 450) among bicycle users. These were followed by pedestrian collisions and, less frequently, by collisions with other vehicles. When analysing only the 16 cases classified as severe (with an ISS ≥ 16), most also resulted from single-vehicle crashes (N = 10; 62.5%) (Figure 3 and Figure 4).

Helmet use was documented in only 21 out of 302 cases (N = 21; 1.3% of all cases) with available data, representing approximately 7%. Most accidents involved a single injured individual (N = 1345; 81.3%). The accident location was recorded in just 96 cases (N = 96; 6.1%), with roadways being the most frequent setting (N = 55; 57.3%).

**Figure 5:**
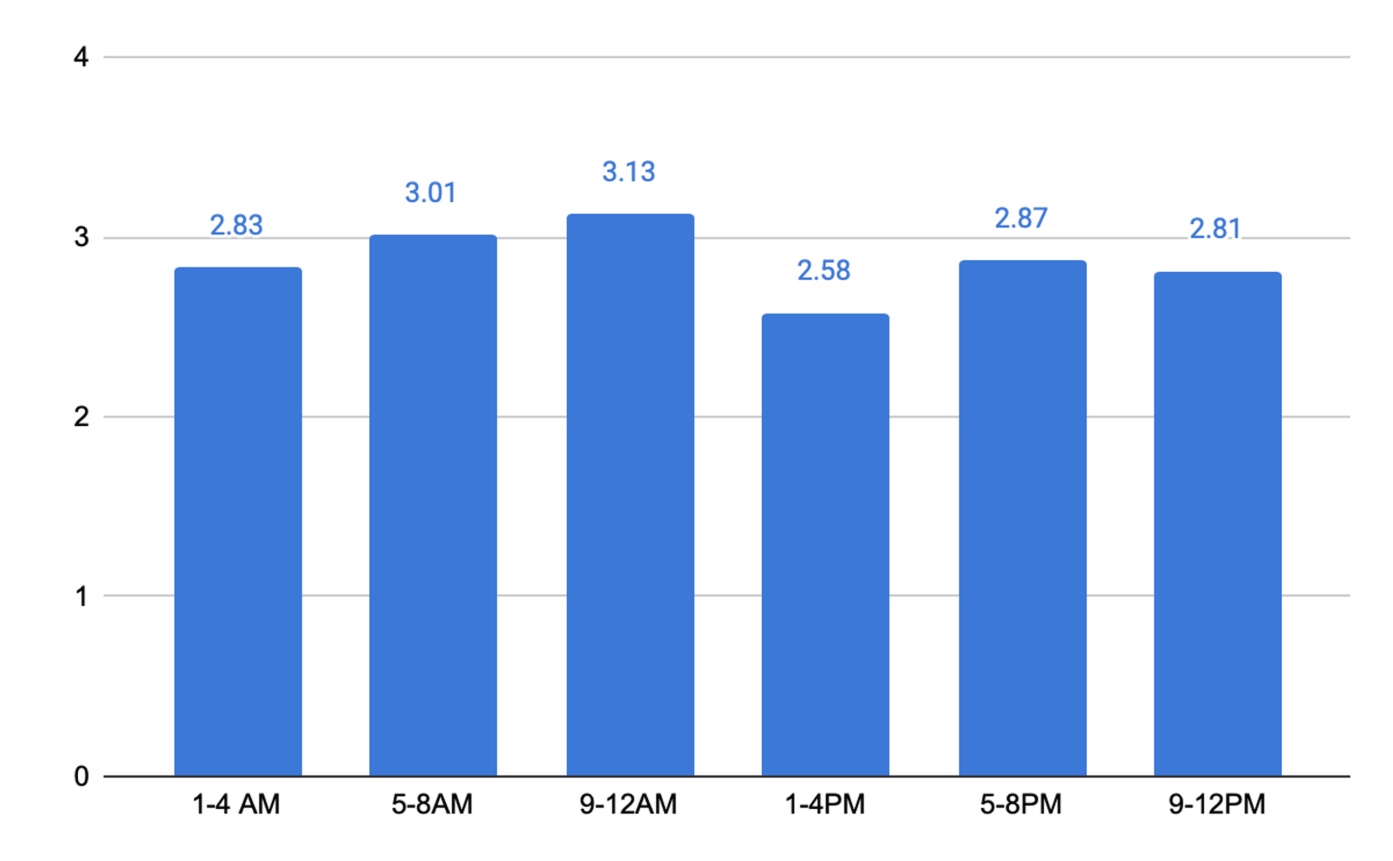
Trauma severity by time of day, according to the Injury Severity Score. The y-axis represents numbers.

Accidents occurred predominantly between 5:00 p.m. and 8:00 p.m. (N = 293; 17.7%) and between 1:00 a.m. and 4:00 a.m. (N = 317; 19.2%), suggesting patterns linked to commuting and recreational mobility, respectively. However, no statistically significant difference in trauma severity was observed between daytime and nighttime periods, with mean ISS values of 2.87 and 2.85, respectively (Figure 5). 

Injuries and severity

**Figure 6:**
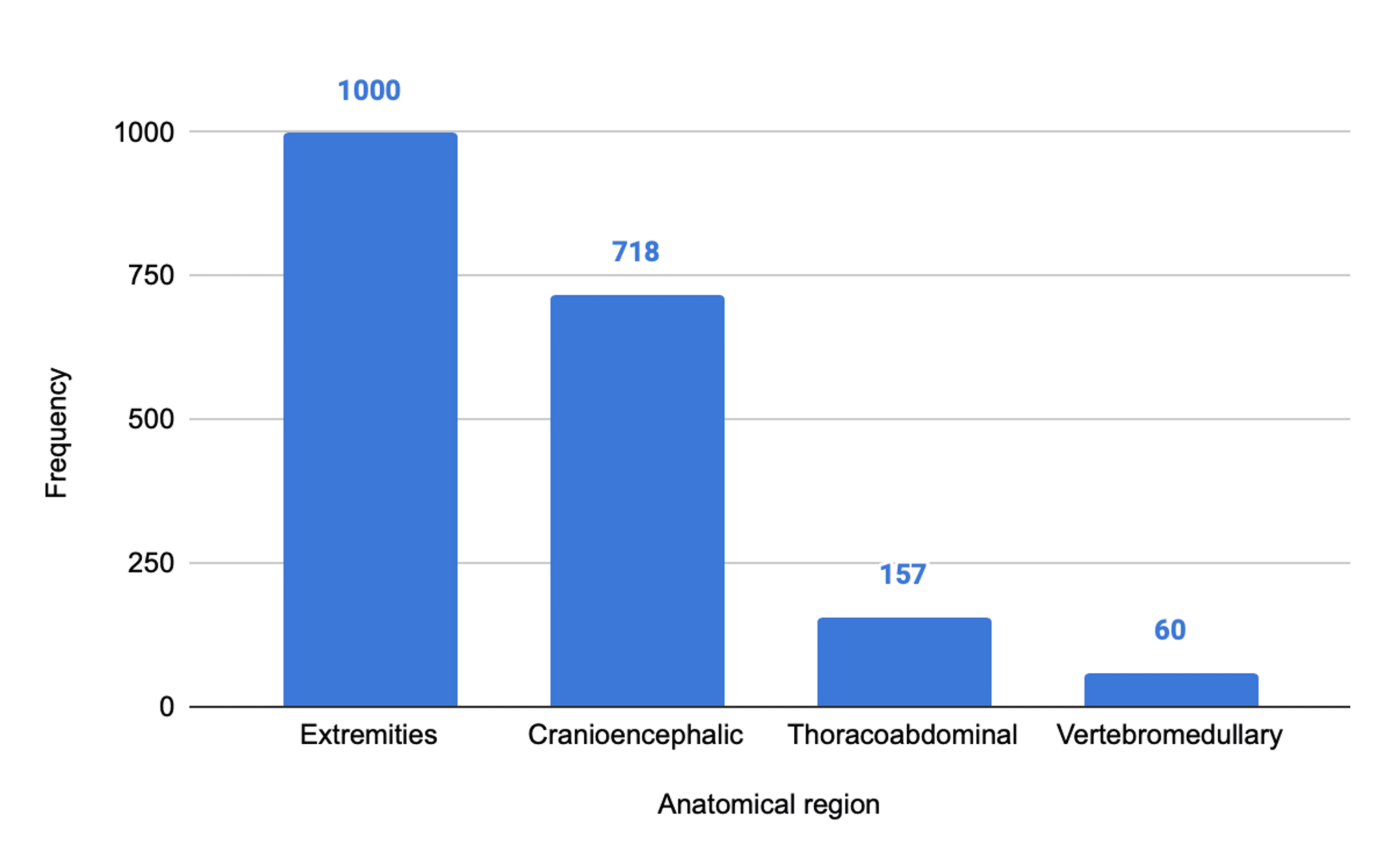
Anatomical distribution of injuries in the general population. The y-axis represents numbers.

**Figure 7:**
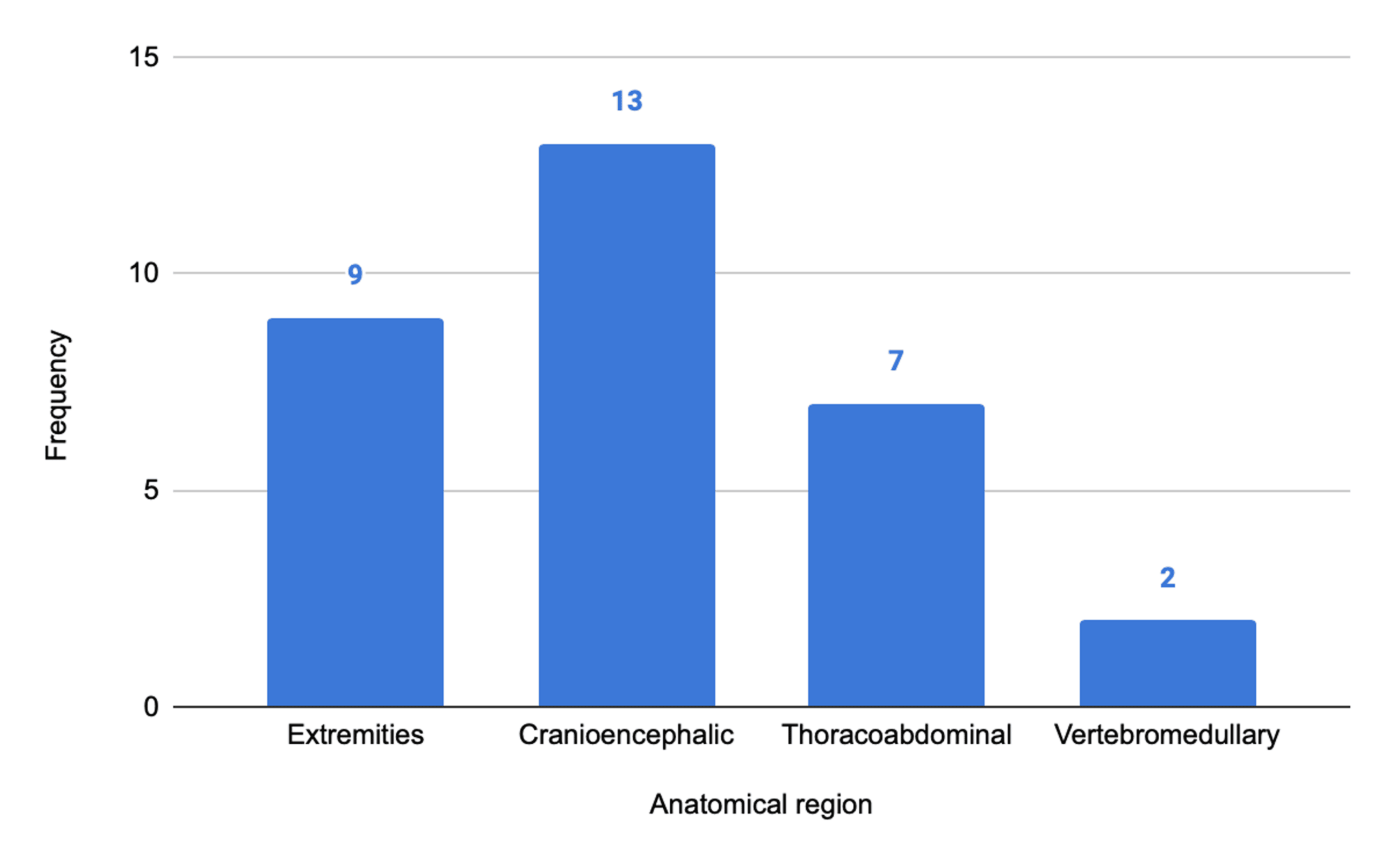
Anatomical distribution of injuries in the severe subgroup (ISS ≥ 16). ISS, Injury Severity Score The y-axis represents numbers.

The most frequent injuries involved the extremities (N = 1000; 51.4%), followed by cranioencephalic trauma (N = 718; 36.9%), thoracoabdominal injuries (N = 167; 8.6%), and vertebromedullary injuries (N = 60; 3.1%) (Figure 6). Among the cases classified as severe, there was a higher proportion of cranioencephalic injuries, as illustrated in Figure 7.

**Figure 8:**
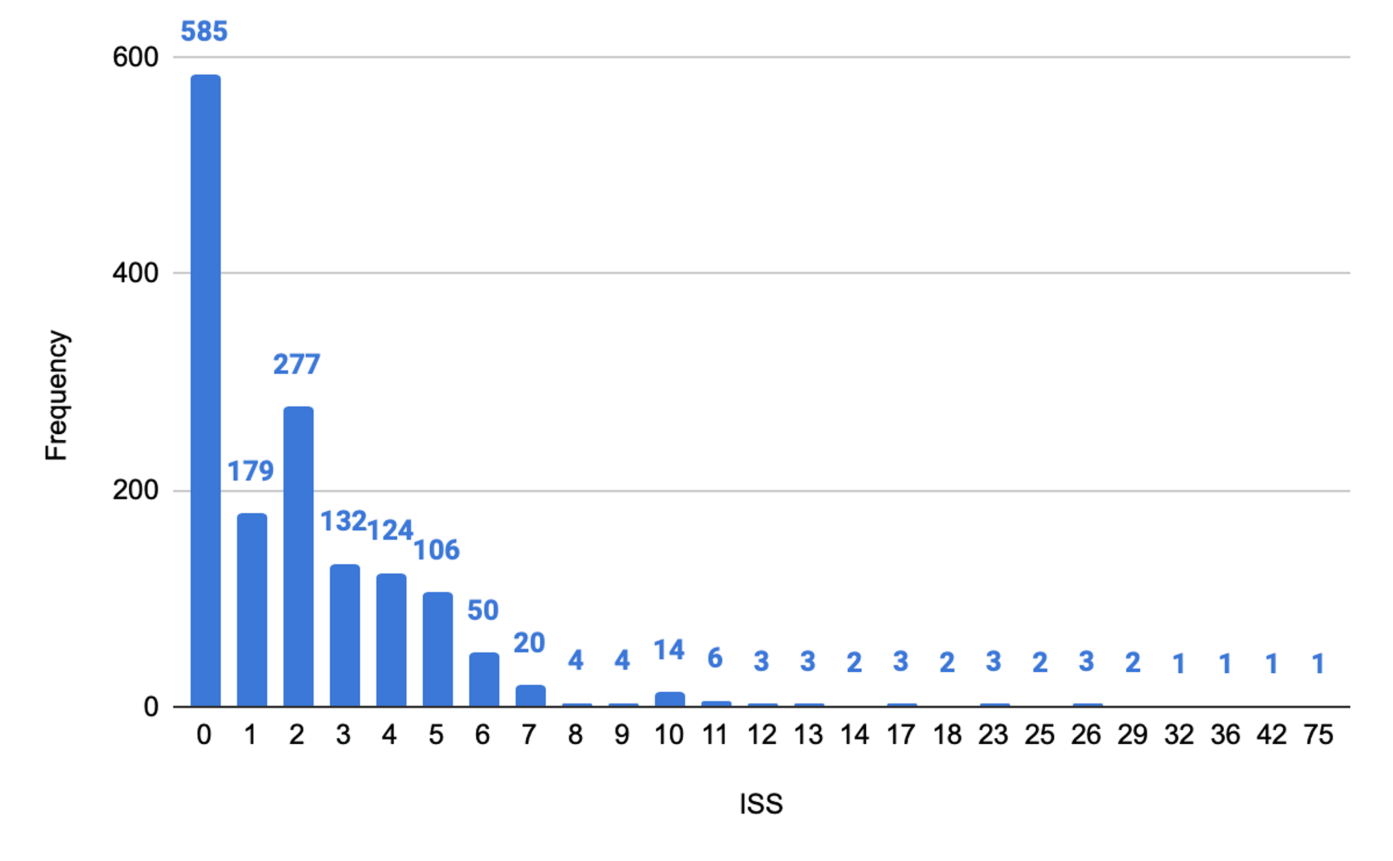
Distribution of trauma severity according to the ISS. ISS, Injury Severity Score The y-axis represents numbers.

The mean ISS was 2.67, with a median of 2, a mode of 1, and values ranging from 1 to 75. The first, second (median), and third quartiles were 1, 2, and 3, respectively. The distribution was right-skewed, reflecting a predominance of minor injuries, although severe trauma cases were also recorded (Figure 8).

Alcohol and other substance use

Ethanol consumption was confirmed in 81 out of 109 tests performed (5.2% of all cases). The presence of other psychoactive substances was infrequent: four cases (N = 4; 0.3% of all cases) tested positive for cannabinoids, three (N = 3; 0.2% of all cases) for opioids, three (N = 3; 0.2% of all cases) for benzodiazepines, and two (N = 2; 0.1% of all cases) for cocaine. Toxicology screening was not performed in 1,520 patients (97.1%).

In inferential analysis, the Mann-Whitney U test revealed a statistically significant difference between the groups with and without alcohol consumption (p = 0.009), with higher ISS values in patients who tested positive for ethanol. Thus, despite the low number of cases, a statistically significant association was found between alcohol consumption and injury severity. No significant differences in trauma severity were observed with other substances; however, the low number of positive screenings limits the robustness of these conclusions.

Resource utilization

A total of 271 patients (17.3%) were hospitalised, with a mean ISS of 6.0 ± 5.3. Among them, 18 (6.6%) required intensive care, with a mean ISS of 10. During hospitalisation, 129 (47.6%) patients underwent at least one surgical procedure, with seven patients (N = 7; 2.6%) undergoing more than one intervention. Despite the high number of surgeries, transfusion of blood products was uncommon, being necessary in six cases (N = 6; 2.2%), including one patient who received multiple units during two procedures.

The mean hospital length of stay was seven days (median: 2; maximum: 215 days). Among ICU patients, the average APACHE II score was 11.1, while the mean SAPS II and SAPS III scores were 35.6 and 57.5, respectively. The average ICU stay was 7.2 days (median: 2.5 days). Based on the rates from Ordinance no. 163/2013, hospitalisations are estimated to have resulted in a total cost of approximately €456,155.44, or about €1,683.20 per hospitalised patient. This includes roughly €388,734.50 for general ward stays (253 patients, 7 days on average) and €67,420.94 for intensive care unit admissions (18 patients, 7.2 days on average per episode). Of the patients admitted to the ICU, seven (N = 7; 38.9%) belonged to the severe trauma subgroup [12].

During hospitalisation, 433 patients (27.7%) underwent at least one computed tomography (CT) scan, and 157 (10%) had two or more. According to ordinance no. 173/2024, the average cost of a CT scan is €73.2, bringing the estimated total cost of imaging to €31,695.60 [13].

Primary and secondary outcomes

**Figure 9:**
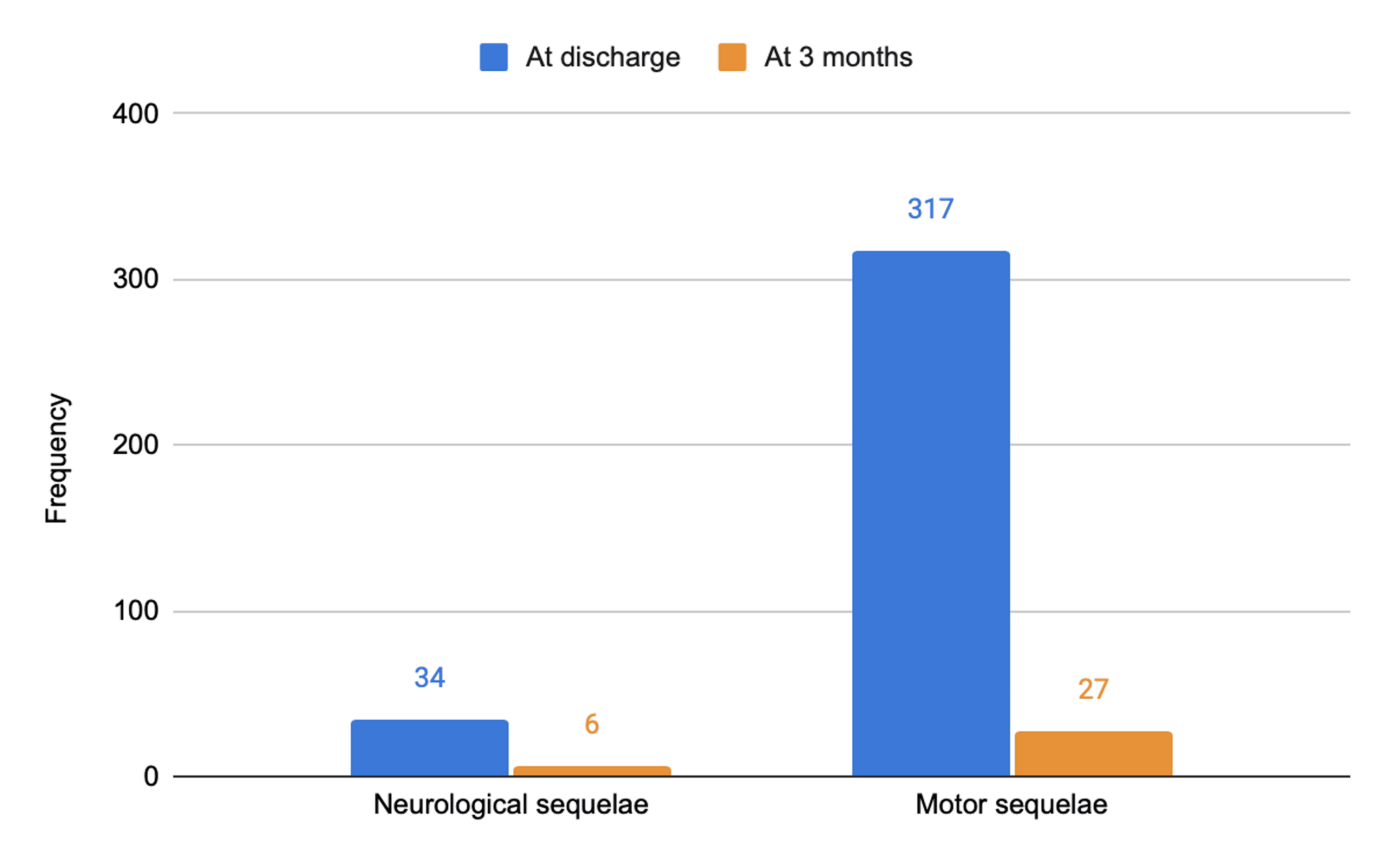
Evolution of neurological and motor sequelae at discharge and after three months. The y-axis represents numbers.

At discharge, 1,377 patients (87.9%) were discharged home, 82 (5.2%) were transferred to community institutions, 80 (5.1%) to other hospitals, and there were five (0.3%) in-hospital deaths. Motor sequelae were documented in 317 patients (20.2%) and neurological sequelae in 34 (2.2%). Three months after discharge, the number of patients with motor sequelae dropped to 27 (1.7%) and those with neurological sequelae to six (0.4%), as illustrated in Figure 9.

Work or school disability was documented in 48 cases (3.1%) lasting less than one month, in 34 cases (2.2%) between one and three months, and in 11 patients (0.7%) for more than three months.

## Discussion

This study aimed to comprehensively describe the clinical, epidemiological, and economic characteristics of injuries associated with micromobility vehicles admitted to a Portuguese tertiary hospital over five years. The majority of victims were of foreign nationality. Given the diversity of nationalities and the inability to distinguish between residents and non-residents, different patterns of usage and, consequently, of accidents may exist, so we cannot determine whether this is related to an actual increase in accidents.

Most accidents involved electric scooters, reflecting a growing trend in the use of this transport mode in urban areas, consistent with global patterns. The low rate of helmet use (7% of cases with available data) and the high proportion of alcohol use (5.2% of tested cases) highlight frequent risk behaviours. The lack of systematic recording of helmet use and toxicology testing is an important limitation for data interpretation and further emphasises the need for structured trauma registries that enable broader public health analysis and planning. Nonetheless, the findings are consistent with prior studies identifying the absence of helmet use as an independent risk factor for traumatic brain injury. The results of MaTrICS distribution of accident mechanisms involving electric scooters, when compared to bicycles, suggest that specific characteristics of scooters may increase the risk of single-vehicle crashes, whereas bicycle-related trauma appears to be more commonly associated with interactions involving motorised traffic [12-16].

Although minor injuries predominated (median ISS = 2), a subset of patients presented with severe trauma, requiring intensive care and resulting in long-term functional impairment, including work-related disability in 93 individuals. Alcohol use was significantly associated with higher injury severity (p = 0.009), consistent with international literature linking alcohol consumption to both accident risk and injury severity in micromobility contexts [17]. However, this association is limited by the fact that toxicology screening was often only performed in more serious cases. No conclusions can be drawn regarding other substances due to their low incidence, likely a result of low testing rates.

The hospital burden associated with these accidents was considerable. Over 270 patients required hospitalisation, including 18 admitted to the ICU. Direct healthcare costs related to hospital stays and CT scans exceeded €480,000, based on unit costs defined by Portaria no. 163/2013 and Portaria no. 173/2024 [12, 13]. These figures align with one of the main goals of this study - to quantify the economic burden of micromobility-related trauma in Portugal, a topic still underexplored and likely underestimated due to the absence of systematic trauma admission records. This trend was previously reported in Spain [6].

A considerable number of patients presented with neurological or motor sequelae at hospital discharge, although most showed significant recovery by three months. The functional impact - in conjunction with the need for ICU admission, surgery, and imaging - must be considered in assessments of the overall burden of disease and in the design of prevention, urban planning, and road safety strategies.

Among severe trauma cases (ISS ≥16), a small number of patients were identified, though the clinical and functional consequences were substantial. Most of these cases resulted from isolated falls, underscoring that seemingly minor events - such as short-distance scooter rides - can lead to life-threatening injuries, especially when protective equipment is not used. The most frequent injuries in this group were traumatic brain injuries. These findings highlight the disproportionate healthcare resource use associated with this subgroup. Therefore, micromobility must be recognised as a potential cause of major trauma, justifying the implementation of public health measures - including helmet promotion, alcohol use prevention, and the development of safe urban infrastructure - even for short urban commutes.

Limitations

This study has limitations inherent to its retrospective design and the lack of a structured trauma admission registry, particularly regarding variables such as helmet use and accident location. In minor trauma cases, toxicology testing was frequently omitted, limiting the robustness of related conclusions. Nevertheless, the large number of cases included and the detailed clinical, functional, and economic characterisation represent key strengths, providing a valuable basis for future public health policy discussions in this area.

## Conclusions

To the best of our knowledge, this is the first nationwide study to describe trauma and its associated costs from micromobility-related accidents in Portugal. Although most cases involved minor injuries from scooter accidents, a minority resulted in severe trauma, with significant morbidity, mortality, and economic impact. The suggested associations with alcohol use and lack of helmet use warrant further investigation. There is an urgent need to implement preventive measures and establish prospective standardised registries to enable continuous monitoring and inform effective public policy.
